# Effects of assisted oocyte activation with calcium- ionophore and strontium chloride on *in vitro* ICSI outcomes

**DOI:** 10.22038/IJBMS.2018.30422.7331

**Published:** 2018-11

**Authors:** Marziyeh Norozi-Hafshejani, Marziyeh Tavalaee, Leila Azadi, Mehrnoosh Bahadorani, Mohammad Hossein Nasr-Esfahani

**Affiliations:** 1 Department of Reproductive Biotechnology, Reproductive Biomedicine Research Center, Royan Institute for Biotechnology, ACECR, Isfahan, Iran; 2 Department of Biology, Falavarjan Branch, Islamic Azad University, Isfahan, Iran; 3 Isfahan Fertility and Infertility Center, Isfahan, Iran

**Keywords:** Fertilization, Implantation, Ionomycin, Pregnancy, Strontium

## Abstract

**Objective(s):**

Failed fertilization after intra-cytoplasmic sperm injection (ICSI) is mainly attributed to failed oocyte activation and can be overcome by artificial oocyte activation (AOA). The present study aims to compare *in vitro* outcomes of ICSI following two different assisted oocyte activation chemical procedures (SrCl_2_ and Ionomycin) in sibling oocytes of ICSI candidates.

**Materials and Methods:**

From March 2015 until February 2016, 105 infertile men with 99–100% abnormal sperm morphology, irrespective of sperm motility, concentration, or origin (semen or testicular) were included in this study. Out of these, 66 couples accepted to be included in the study group (Ionomycin/ SrCl_2_) and 39 couples requested routine AOA procedure (Ionomycin) as external control group. Primary outcomes of this study (fertilization, embryo quality, and post-implantation development) were compared between these groups.

**Results:**

Significantly higher oocyte activation (67.90±3.6% vs. 51.16±3.6%, *P*=0.004) and fertilization (65.23±3.63% vs. 49.65±3.63%, *P*=0.008) rates were observed in sibling oocytes treated with Ionomycin in comparison to the SrCl_2_ sibling group. Percentage of top quality embryos was insignificantly higher in SrCl_2_ groups compared to the Ionomycin group (29.90±4.27 vs. 20.65±4.05%, *P*=0.26).

**Conclusion:**

Ionomycin may be superior to SrCl_2_ for inducing oocyte activation. However, SrCl_2_ may be a more efficient means to support the development of better quality embryos following ICSI.

## Introduction

Since its introduction in the early 1990s, intracytoplasmic sperm injection (ICSI) has been increasingly used as the most successful assisted reproductive technique (ART) in cases of severe male factor infertility (1). The outcomes of ICSI in terms of fertilization, embryo transfer, and clinical pregnancy rates are rewarding and are either greater or equal to the corresponding rates of conventional *in vitro* fertilization (IVF) (2). 

Nonetheless, failed fertilization occurs in up to 3% of ICSI cycles (3). Owing to the fact that ICSI subverts many steps of natural fertilization and almost any type of sperm may be used for ICSI, failed fertilization after ICSI is theoretically attributed to a number of factors including oocyte spindle defects, premature sperm chromatin condensation, failure in sperm head decondensation, sperm aster defects, and severe defects in sperm morphology and DNA integrity. However, several lines of evidence indicate that the main cause of failed fertilization after ICSI is the inability of injected sperm to induce intracellular calcium release and meiosis resumption of MII-oocyte, a process called oocyte activation (4, 5). 

It is well established that a testis-specific, sperm-borne oocyte activating factor (SOAF) is responsible for the activation of oocytes during normal fertilization (6). Among several SOAF candidates, phospholipase C zeta (PLCζ) fulfills crucial characteristics of SOAF (6-8). Studies have demonstrated a link between PLCζ deficiencies with failed fertilization, particularly in men with globozoospermia (9-13). On the other hand, associations between SOAFs with sperm quality and fertilization ability have been reported in an increasing number in recent studies (6, 14, 15). It seems that reduced level of these factors, in some semen samples, is associated with reduced ability to induce oocyte activation post-ICSI, and therefore, there is a need for effective artificial activation procedures following ICSI.

It is well established that MII oocytes of almost invariably all mammalian species respond to artificial activation by representing calcium oscillations which are closely similar to those induced during natural fertilization (16). Among different chemical, electrical, and mechanical protocols devised for assisted oocyte activation (AOA) (17-20), chemical-AOA is increasingly used in assisted reproductive clinics as an efficient method to restore fertilization in couples with a history of failed fertilization (21-25). Nonetheless, there is no consensus regarding the type of chemical agent that should be used for AOA. Importantly, the only commercially available media for AOA is Ca-ionophore (26). 

Mortimer *et al.* (1986), for the first time, showed that the rate of human sperm penetration into the hamster oocytes was higher in sperm population pretreatment with media containing SrCl_2_ compared to calcium-containing media (27,28). In addition, studies in mice, as the most studied system, suggested superiority of SrCl_2_ for oocyte activation when compared to Ca-ionophore (29). Furthermore, a study suggested that calcium oscillations induced by SrCl_2_ are more similar to those provoked during natural fertilization in mice when compared to Ca-ionophore (30). Another study in mice found no gross differences between the efficacies of SrCl_2_, Ca-Ionophore, and electrical pulse for mouse AOA, although the pregnancy rates were in favor of SrCl_2_ and electrical pulses (16). 

Mechanistically, sperm-mediated oocyte activation is usually induced via a two-step pattern of rises in intracellular calcium concentrations. The first rise, which is a tonic rise in calcium concentration originates from the oocyte cortex and is induced immediately after sperm-oocyte membrane interaction. This tonic rise is followed 30 min later by several oscillations in intracellular calcium concentrations that continue for 3–4 hr (31). Calcium ionophore induces a singular tonic rise in Ca^2+^while SrCl_2_ can induce few to several Ca^2+^ transients similar to physiologic patterns of Ca^2+^ release in mice (32). To our knowledge, there is no clinical study for comparison of pre- and post-implantation development of human ICSI oocytes following AOA by SrCl_2_ and Ca-ionophore. Therefore, this study compared *in vitro* outcome of ICSI following two different assisted oocyte activation protocols in infertile men with abnormal sperm morphology or azoospermic individuals that were candidates for testicular sperm aspiration (TESA). 

## Materials and Methods

***Study design***

This study was approved by the Ethical Committee of Royan Institute (IRCT201510257223N7; 2/12/2015) and all patients provided written informed consent for their participation and usage of their information and data. 

AOA using Ca-ionophore is routinely offered to infertile couples with abnormal sperm morphology ranging between 99–100% and azoospermic individuals that are candidates for testicular sperm aspiration (TESA). Therefore, the basis of patient selection in the current study for AOA was previous studies in this field. (17,33-36). 

***Study population***

**Figure 1:**
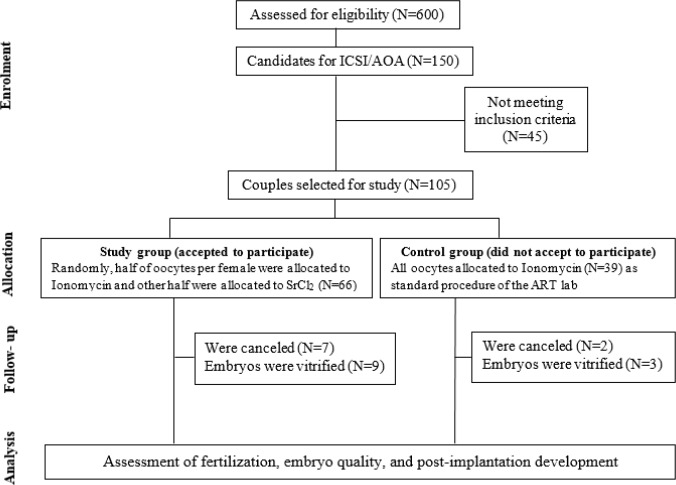
Flow diagram of study design

Of 600 infertile couples referring to Isfahan Fertility and Infertility Center (IFIC) from March 2015 until February 2016, 105 couples had the inclusion criteria of this study. Out of 105 couples, 66 couples agreed to be included in the study group (Ionomycin/ SrCl_2_) while 39 couples requested the routine AOA procedure using Ionomycin and were considered as the external control group. The final patient population in the study group consisted of 19 (28.9%) and 47 (71.2%) couples, candidates for TESE-ICSI and ICSI, respectively. The external control group consisted of 15 (38.5%) and 24 (61.5%) couples, candidates for TESE-ICSI and ICSI, respectively (Figure 1). Couples candidate for routine ICSI provided semen samples on the day of ICSI. Frozen TESE samples were used for TESE–ICSI cases. In this study, mean ages of female (29.43±00.68 vs. 30.15± 00.94) and mean ages of men (34.70±00.79 vs. 35.46± 01.43) were not significantly different between study and control groups (Table 1).

***Exclusion criteria***

In the IFIC center, females older than age 45 are not included in ART procedures. Individuals with leukospermia, urinary infection, Klinefelter syndrome, cancer, and excessive alcohol or drug abuse were excluded. To reduce female confounding factors, couples with female factor infertility or couples having less than 6 matured oocytes, poor quality oocytes (abnormal zona pellucida, large perivitelline space, refractile bodies, increased cytoplasmic granularity, smooth endoplasmic reticulum clusters, or abnormal, fragmented, or degenerated polar bodies) and endometrial thickness less than 7mm or with type C endometrium were excluded from this study. Accordingly, only couples with male factor infertility were included in this study. 

***Study intervention***

ICSI-AOA candidate couples were counseled regarding the AOA procedure, type of chemicals (Ca-ionophore and SrCl_2_), and the clinical trial. Couples who accepted to be included in this clinical trial were considered as the study group. In the study group inseminated oocytes after ICSI, were randomly divided into two groups and AOA was carried out using either Ca-ionophore (sibling Ca-ionophore-AOA) or SrCl_2_ (sibling SrCl_2_-AOA). Randomization of inseminated oocytes to Ca-ionophore and SrCl_2_ were carried out by the embryologist, who was blind to the study. After AOA, the dishes were randomly numbered so that the embryologist assessing the ICSI outcomes (fertilization and embryo quality) was also blind to the study. The couples who did not agree to be included in the study and requested routine treatment (Ca-ionophore-AOA) but permitted us to use their clinical information were considered as the external control group. ICSI oocytes of this group were only treated with Ca-ionophore (Figure 1) and the same embryologist who was blind to study design assessed the ICSI outcomes**.** The Ca- ionophore used in this study was Ionomycin (19). Primary outcomes of this study were fertilization rate, embryo quality, and post-implantation development, which were compared between study and control groups.

Out of 66 ICSI cycles performed in this study, 7 cycles were canceled. These couples had either no fertilization or no suitable embryos for transfer. Data of these 7 cases were only included for assessment of fertilization and oocyte activation, but not for assessment of implantation and pregnancy rates. Out of the remaining 59 cases, all embryos of 16 cases were vitrified. Seven of these 16 cases (3 from Ionomycin and 4 from SrCl_2_) referred for transfer of their frozen embryo and all embryos of the remaining 9 cases are still vitrified. Since the implantation and pregnancy rates of fresh and vitrified/warmed cycles in our center are very similar, the results of these 7 cases were also included for assessment of implantation and pregnancy rates. Out of 39 ICSI cycles performed in this study, 2 cycles were canceled and all embryos of the remaining 3 cases are still vitrified (Figure 1).

***Semen analysis***

Semen analysis was carried out by light microscopy according to the World Health Organization (WHO) criteria. Concentration, motility, and morphology of sperm samples were assessed by sperm processor (Sperm meter, Aurangabad, India), computer-assisted sperm analysis (CASA), and Papanicolaou staining, respectively (37).

***Sperm processing (sperm density-gradient washing)***

Semen samples were washed and diluted in 1 ml of VitaSperm (Inoclon, Iran) supplemented with 10% HSA (Human Serum Albumin, OctaPharma). Samples were then layered over discontinuous gradients (90% and 45%) of PureSperm (Nidacon, Sweden) and centrifuged at 1000 rpm for 15 min. Subsequently, the pellet was washed twice with VitaSperm plus 10% HSA and diluted in the same medium. TESE samples were washed three times with VitaSperm plus 10% HSA.

**Figure 2:**
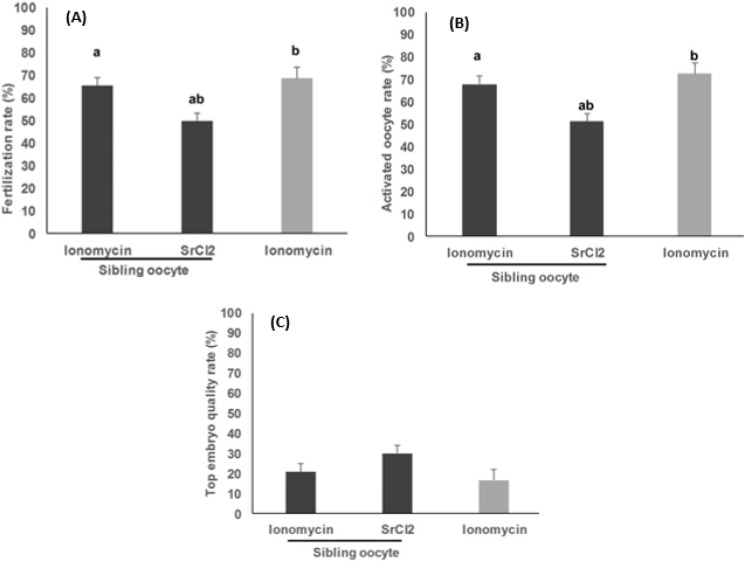
Comparison of the clinical outcomes after ICSI [fertilization (A), activated oocyte (B), and top embryo quality (C) rates] between the sibling oocytes undergoing artificial oocyte activation using Ionomycin and/or strontium chloride. the results were also compared with an external group undergoing artificial oocyte activation using only Ionomycin

Common letters showed significant difference at *P*<0.05

**Table 1:** Mean of semen parameters and couples’ characteristics in the study and external control groups

Parameters	**Study group** **66 cases**	**External control group** **39 cases**	***P*** **-value**
	**Mean + SE**		
**Male age**	34.70 ± 00.79	35.46 ± 01.43	0.61
**Female age**	29.43 ± 00.68	30.15 ± 00.94	0.53
**Sperm concentration (10** ^6^ **/ml)**	18.66 ± 04.51	16.96 ± 04.55	0.83
**Total sperm motility (%)**	23.43 ± 02.56	21.36 ± 03.27	0.62
**Total oocytes**	14.46 ± 00.85	12.71 ± 00.87	0.08
**Total injected oocytes**	11.68 ± 00.64	10.25 ± 00.78	0.16
**Previous ART**	00.58 ± 00.11	00.67 ± 00.13	0.59
**Duration of infertility (years)**	5.82 ± 00.60	6.02 ± 00.73	0.83

**Table 2:** Assessment of clinical parameters in this study

**Parameters**	**Study group (sibling oocytes)** **N=66**			**External control group** **N=39**
	**Ionomycin** **number (percentage)**	**SrCl**_2_ **number (percentage)**	**Ionomycin** **& SrCl**_2_ **number (percentage)**	**Ionomycin** **number** **(percentage)**
Mean transferred embryos	1.91±0.15^a^	2.38±0.15^ab^	1.83±0.15	2.02±0.08^b^
Mean number of transferred embryo(s) per transfer	1.68±0.15^a^	1.85±0.15^b^	2.69±0.2	2.26±0.12^ab^
Number of fresh transferred embryo(s) + frozen transferred embryo(s)	13+3 (24.24)	17+4(31.81)	10+3(19.69)	27+7 (87.18)
Number of clinical pregnancy	4/16 (25.00)	5/21 (23.8)	4/13(30.76)	8/34 (23.52)
Implantation rate (sac/no. transferred embryo(s)	6/27 (22.3)	5/41(12.2)	4/35(11.42)	7/77 (9.09)
Abortion	00	1	00	1
Still birth	00	00	00	00
Singletons	2	4	4	7
Twins	2	0	0	0
Male/Female	4/6	2/2	2/2	3/4

Common letters indicate significant difference between samples at *P<0.05.*

***ICSI, embryos culture, embryo transfer, and pregnancy follow up***

All media used for ICSI and embryo culture procedures were purchased from Vitrolife (Gothenburg, Sweden, G5 series plus) unless otherwise stated. After oocyte retrieval, oocytes were initially dissected and then denuded of remaining granulosa cells with hyaluronidase enzyme [Hyase (90IU/ml); Vitrolife, Gotenborg, Sweden] in G-MOPS medium, a pH stable handling medium designed to support the handling and manipulation of oocytes and embryos outside the incubator. Oocytes were then washed and transferred to G- MOPS under oil in a dish prepared for ICSI. Meanwhile, a PureSperm processed semen sample was also introduced into ICSI-100 (a viscous medium for reduction of sperm motility to facilitate sperm handling) prepared in an ICSI dish immediately before the ICSI process being performed. Inseminated oocytes from the study group were randomly divided into two groups (Ca-ionophore and SrCl2)) and at 16–18 hr post-ICSI, fertilization rate was assessed according to the presence or absence of pronuclei (PN). Then, the number of pronuclei was recorded. Oocytes with at least one pronucleus were considered as activated, oocytes with two pronuclei were considered as fertilized and oocytes with more than two pronuclei were considered as abnormally fertilized. Presence of at least one pronucleus was considered a sign of oocyte activation and total percentage of activated oocytes was calculated based on the number of oocytes containing at least one pronucleus per total number of injected oocytes. It is important to note that in literature, the presence of one PN post IVF/ICSI has also been related to parthenogenetic activation due to various possible stimuli (38, 39). In this study, since all oocytes are activated by Ionomycin or SrCl_2_, the presence of one PN is very likely related to chemically induced activation. Therefore, we assessed both normal fertilization and percentage of activated oocytes, to overcome any wrong conclusions**.** In this study, the fertilization rate was calculated by dividing the number of fertilized oocytes (2PN) by the total number of survived injected metaphase II (MII) oocytes multiplied by 100 in two groups. It is important to note the division of inseminated oocytes into two groups was random. Post-insemination assessments of fertilization and embryo quality were also random as the dishes post grouping were randomly numbered. 

Embryo quality was assessed three-days post-oocyte retrieval using a three-point scoring system (18, 40, 41) and taking into account the following parameters: i) absence or fragmentation of < 25% on embryonic surface, ii) equality of blastomere’s size and shape, and iii) blastomere cell number greater or less than 7. Embryos presenting all parameters were scored as “3”, embryos having only 2 parameters were scored as “2” and embryos presenting only one of the parameters were scored as “1”. Mean embryo score was calculated by summing the embryo score in each group divided by the number of embryos scored in each group. Therefore, the mean embryo score could vary from 3 to 1. Score 3 embryos were considered as top quality embryos.

The number of embryos transferred to each patient on day 3 was based on patient age, number of previous failed cycles, quality of embryos at the time of embryo transfer and the final decision made by the gynecologist. Embryo for transfer was based on best embryo quality irrespective of the group. Pregnancy was confirmed by measurement of ß-hCG, and the presence and number of fetal sacs and fetal heartbeat that were defined with the aid of ultrasound. Pregnancy rate was calculated by either the number of pregnancies per ovum pick-up or per embryo transfer cycle.

It is important to note that since there is no regulation for number of embryo transfers in Iran, couples can request to transfer more than one, day 3 embryo. In our center generally a maximum of two embryos on day 3 are transferred. A couple may have more than 2 embryos (3embryos) for transfer on day 3 if A) the female age is greater than 35, B) two previous ICSI cycles were unsuccessful, or C)number of embryos with good quality is low.

***Assisted oocyte activation***

Injected sibling oocytes in the study group were divided into two groups (sibling oocytes): in one group oocytes were treated with 10 M Ionomycin (Sigma; Ca:10634, stock were prepared in DMSO) in the G-MOPS media for 10 min immediately post-ICSI, and the oocytes in the other group were initially incubated at 37 °C and 5% CO_2_ for 30 min post-ICSI and then stimulated with 10 mM SrCl_2_ in GPGD medium (Gothenburg, Sweden, G3 series plus) for 60 min. GPGD is a calcium- and magnesium-free MOPS buffered medium that maintains pH during the AOA procedure and was chosen based on a previously reported procedure (42). In the external control group, injected oocytes were artificially activated by exposure to 10 M Ionomycin in G-MOPS medium for 10 min immediately post-ICSI. The oocytes in each group were randomly labeled so that the embryologist, on the day of scoring, was unaware of the groups’ identities. Following the scoring of embryos developed in the study group, the embryologist was asked to select two good quality embryos from each group and to transfer these embryos. In cases that only one good quality embryo was available in sibling Ca-ionophore-AOA group, the second or third embryo was selected from the sibling SrCl_2_-AOA group or vice versa. For couples with mixed embryos transferred, data were not included for analysis of implantation and pregnancy rates. In the external control group, the embryologist was also advised to select at least one good quality embryo for transfer. 

***Statistical analysis***

Based on the previous studies, samples size for the sibling oocytes was determined to be around 60 (33). For descriptive results, data were expressed as mean ± error of the mean (SEM) except for male and female age and also the duration of infertility. For statistical analysis, Chi-square for clinical pregnancy and implantation rate, Student’s t-test for sperm parameters, age, number of oocytes, number of previous ARTs, and duration of infertility, and one-way analysis of variance (ANOVA) for fertilization, activated oocytes and top embryo quality rate were carried out using the Statistical Package for the Social Sciences software (SPSS 18; Chicago, IL, USA). Differences were considered significant at *P*< 0.05.

## Results

***Patients’ clinical data***

Out of a total of 105 ICSI cycles studied, 66 were included in the study group and the remaining 39 were included in the external control group. Couples’ characteristics and average semen parameters used in the study and external control groups are presented in Table I. All stated parameters including age of couples, duration of infertility, number of previous ART cycles, semen characteristics, and total and injected number of oocytes were similar (*P*>0.05) between the study and external control groups (Table 1). 

***Activation and fertilization rates following ICSI-AOA with Ca-ionophore and SrCl***_2_

Comparison of the clinical outcomes of ICSI combined with AOA between the sibling oocytes in the study group (sibling Ca-ionophore-AOA *vs*. sibling SrCl_2_-AOA) and also between the study and the external control groups are shown in Figure 2.

AOA with Ionomycin significantly increased fertilization rate (65.23±3.63%) compared to AOA with SrCl_2_ (49.65±3.63%) in sibling oocytes of the study group (*P*=0.008). Also, AOA with Ionomycin significantly increased fertilization rate in the external control group compared to the SrCl_2_ group (68.58±4.72% *vs*. 49.65±3.63%, respectively, *P=*0.005). Likewise, Ionomycin-treated oocytes showed a significantly higher oocyte activation rate compared to the SrCl_2_ -treated group (67.90±3.6% *vs.* 51.16±3.6%, respectively, *P*=0.004). Also, oocyte activation rate in the external control group was significantly higher than SrCl_2_-treated group (72.51±4.69 *vs.* 51.16±3.6%, respectively, *P*=0.001).

***Embryo quality following ICSI-AOA with Ca-ionophore and SrCl***_2_

The mean percentages of top quality embryos were 16.70±5.09, 29.90 ±4.27, and 20.65±4.05% in external control, sibling SrCl_2_-AOA, and sibling Ca-ionophore-AOA groups, respectively. Embryo quality of the sibling SrCl_2_-AOA group was higher compared to external control (*P=*0.12) and sibling Ca-ionophore-AOA (*P*=0.26) groups, the observed differences were not statistically significant.

Within the sibling Ca-ionophore-AOA group, 191 embryos showed cytoplasmic fragmentation lower than 25% and 33 embryos showed cytoplasmic fragmentation higher than 25%. Out of 149 embryos assessed in the sibling SrCl_2_-AOA group, 137 embryos showed cytoplasmic fragmentation lower than 25% and 12 embryos showed cytoplasmic fragmentation higher than 25%. Indicating a significant difference in terms of percentage of embryos with high fragmentation rate between the two AOA methods of sibling oocytes (*P*=0.05). 

***Quality of embryos selected for transfer following ICSI-AOA with Ca-ionophore and SrCl***_2_

In this study, we also compared the mean embryo score of transferred embryos between the groups. Mean embryo scores (see methods) in this study were 1.91±0.15, 2.38±0.15, and 2.02±0.08 in sibling Ca-ionophore-AOA, sibling SrCl_2_-AOA, and external control groups, respectively. This value was significantly higher in the sibling SrCl_2_-AOA compared with the sibling Ca-ionophore-AOA and also external control groups (Table 2). 

***Mean number of embryos transferred*** ***following ICSI-AOA with Ca-ionophore and SrCl***_2_

 Mean number of embryos transferred in sibling SrCl_2_-AOA, sibling Ca-ionophore-AOA groups, and external control groups were 1.85±0.15, 1.68±0.15, and 2.26±0.12, respectively. A significant difference was observed for the number of embryos transferred between external control with sibling SrCl_2_-AOA and sibling Ca-ionophore-AOA groups (Table 2).

***Implantation and pregnancy outcomes***

Implantation rates were 9.09, 12.2, and 22.3% in external control, sibling SrCl_2_-AOA, and sibling Ca-ionophore-AOA groups, respectively. This result indicates no significant difference between these groups (Table 2). Percentages of clinical pregnancy in sibling SrCl_2_-AOA and sibling Ca-ionophore-AOA groups (23.8% and 25.00%, respectively) were similar to the external control group (23.52%) with no significant differences between the groups. Furthermore, the status of pregnancies in each group was followed. No apparent differences were observed between the groups in terms of abortion rate, stillbirth, or sex ratio (statistical analysis was not carried out due to small number). All born infants were assessed for their health status according to our previous report (22) and no abnormalities were observed. 

## Discussion

Several clinical studies have indicated AOA combined with ICSI may improve chances of fertilization and pregnancy in couples with a history of low and failed fertilizations following routine ICSI (32, 43). Recently, criteria to perform AOA have broadened to infertile men with severe sperm defects (18, 19, 33, 34, 44-46). Accordingly, numerous clinical studies have been carried out to evaluate the effectiveness and to a certain degree, safety, of the ICSI-AOA procedure (22, 23, 25). 

In the sibling oocytes of the study group, our results indicate that AOA with Ionomycin significantly increased activation and fertilization rates compared to AOA with SrCl_2_. Similarly, rates of oocyte activation and fertilization were significantly higher in the external control group compared to SrCl_2_. Therefore, to our knowledge, this is the first comparative study in humans suggesting that AOA of ICSI oocytes with Ionomycin has a higher ability to induce oocyte activation compared to SrCl_2_. However, in a limited case report study, Kim *et al.* (2014) showed that SrCl_2_ might be a better agent than ionophore for induction of oocyte activation in humans, the basis of which is the mice model. In addition, they further concluded that the physical and mental status of these children were normal until 72 months after birth (47).

A study demonstrated that calcium oscillations induced by SrCl_2_ are mediated through the IP3 receptor and required PLCζ activation and synergistic action of IP3 (48). SrCl_2_ is thought to move into oocyte down the concentration gradient, causing calcium to be released from the endoplasmic reticulum (48, 20). This may suggest that SrCl_2_ would better mimic the natural Ca^2+^ release wave of sperm-mediated oocyte activation. Therefore, lower ability of SrCl_2_ to induce AOA may be related to reduced expression or altered activity of PLCζ in these individuals. Therefore, based on this information, SrCl_2_ should not be used for individuals with total absence of PLCζ. However, based on literature, a certain percentage of sperm in semen population may lack PLCζ, which may vary with the severity of male infertility (13, 49-51) and this might be the reason for application of AOA in severe male factor infertility. Therefore, based on the results of this study and previous literature, one may suggest until the full mechanism of SrCl_2_ is not elucidated in human sperm, Ionomycin is advised until natural agents like recombinant PLCζ are available for AOA. On the other hand, the presence of low levels of PLCζ has been reported in round head sperm derived from globozoospermic individuals (9,52) or wobbler mouse model for this syndrome (53), even though fertilization following ICSI in these couples or animal model fails due to failure of oocyte activation and this dearth can be overcome by AOA (45). 

Another point to consider in this study is the difference of duration of exposure of oocyte to chemicals [SrCl_2_ (30 min) and Ionomycin (10 min)] and type of media used (Ionomycin was dissolved in medium with calcium while SrCl_2_ was dissolved in calcium-free medium). It is important to note that our aim was not establishing an AOA procedure but was rather to compare the two existing established protocols based on the literature. Despite different durations of exposure, the lower activation rate of SrCl_2_ could be due to the fact that Ionomycin could have led to the usage of external Ca^2+^ whereas SrCl_2_ fully relies on internal Ca^2+^ storages. Another reason for the difference between the two protocols could be the absence of Ca^2+^ in the SrCl_2_ medium and its presence in the Ionomycin medium. It is important to note that SrCl_2_ must be used in a calcium-free medium base, as Ca^2+ ^competes with SrCl_2, _while Ionomycin should be used in medium containing calcium (54). Indeed, initial studies have shown that SrCl_2_ was more effective in activating mouse eggs in a calcium-free medium rather than a calcium-containing medium (29). 

The results obtained in our study on humans appear to be different from the conclusion of these authors based on the mouse model (16, 32). In addition, SrCl_2_ treatment in mice following ICSI has been shown to maintain chromosomal integrity (55). Recently, it was demonstrated that AOA with Ionomycin was more efficient than A23187 in mouse and human oocytes (56). These differences might be due to species differences and according to some reports, mice may not be a suitable model for human, especially for reproductive studies. It was mentioned that the cow is considered as a more suitable model (57, 58). This species difference might be related to inheritance of sperm centriole–centrosome complex. In mice, this complex is destroyed and cytoplasmic asters contribute to the formation of meiotic and mitotic spindles, while in all other mammalian species sperm centriole–centrosome complex is crucial for fertilization and provides dominant material for formation of sperm aster and subsequent mitotic spindles (59). Therefore, the paternal factor cannot account for the differences observed between the siblings groups, as the same source of sperm was used for both groups. In bovines, similar to our study, Ionomycin appears to a stronger agent for induction of oocyte activation than other agents (60).

One strong aspect of our study is the usage of sibling oocytes in humans, which overcomes many of the confounding factors such as male paternal factors that make it difficult to draw a final conclusion. It is interesting to note that Kim *et al.* (2014) reported successful pregnancy in couples with repeated low fertilization rates after AOA with SrCl_2 _(47). However, in another study calcium oscillations in human oocytes activated with SrCl_2 _were not observed (61).

Another important point to be considered in this study, compared with previous studies using SrCl_2_, is the difference in patient selection. In our study, we used AOA for patients with severe male infertility while Kim *et al.* (2014) used from SrCl_2 _for couples with repeated low fertilization that previously were treated with calcium ionophore (47). Other authors also used SrCl_2 _for patients with previous low fertilization rate (32, 62, 63). It is interesting that in very few of our cases, we had a higher rate of oocyte activation and fertilization in the SrCl_2 _group compared to Ionomycin. 

Therefore, the conclusion made by these authors may stand true for previous cases with repeated low fertilization or patients with repeated low fertilization post-treatment with calcium ionophore. However, in general, our results reveal that the oocyte activating capacity of Ionomycin is higher than SrCl_2_. These differences warrant further comparison of SrCl_2 _and calcium ionophores in a selected group of patients with repeated low fertilization or those with repeated low fertilization following ionophore treatment. 

Comparison of mean embryos score in sibling SrCl_2_-AOA and Ca-ionophore-AOA revealed no significant difference between the two groups. However, comparison of top quality embryos revealed a higher incidence of top quality embryos in SrCl_2_-AOA rather than Ca-ionophore-AOA. Furthermore, mean embryo scores of embryos transferred was significantly higher in sibling SrCl_2_-AOA compared to sibling Ca-ionophore-AOA. It has been suggested that high cytosolic free calcium level induces H_2_O_2 _generation which leads to egg apoptosis through a mitochondrial-caspase-mediated pathway which appears as cytoplasmic fragmentation (64). 

Therefore, in addition to three previous parameters applied for embryo quality scoring (cleavage stage, equality of blastomeres, and degree of cytoplasmic fragmentation), in this study, we also compared the percentage of embryos with lower and higher than 25% cytoplasmic fragmentation. This latter assessment shows a significant difference between sibling SrCl_2_-AOA and sibling Ca-ionophore-AOA, and suggests increased cytoplasmic fragmentation observed in sibling Ca-ionophore-AOA may be due to a single tonic calcium provoked rise; while SrCl_2_ provokes a series of milder but longer Ca^2+^ transients leading to calcium oscillations similar to normal fertilization. Taken together, these data suggest that despite higher rates of oocyte activation and fertilization obtained by Ca-ionophore-AOA, the quality of embryos derived from SrCl_2_ appears to be higher than those of the ionophore group, in terms of cytoplasmic fragmentation. 

The main aim of this study was to assess the ability of two different chemical protocols: SrCl_2_ and Ca-ionophore to induce artificial oocyte activation and to assess the quality of embryos between the two groups, but not the assessment of pregnancy outcomes. In order to compare the effects of these compounds on pregnancy parameters such as implantation and abortion rates, we need a substantially higher number of cases to increase the power of the study. Moreover, we could not carry out randomization during embryo transfer, and embryos were merely selected based on the presence of top quality embryos in each group. The pregnancy outcomes of this study revealed no difference between the two sibling groups and the external control group. 

**Conclusion**

## Conclusion

Results of this study, as a first trial in humans, suggest that Ca-ionophore may be more efficient in inducing oocyte activation compared to SrCl_2_. However, this higher activation ability may perturb the subsequent quality of embryos derived by Ionomycin-AOA. Further studies are needed to compare clinical outcomes of ICSI simultaneously between sibling oocytes that were activated with Ca-ionophore or SrCl_2_ and those without any treatment in a large population. 
